# Endobarrier as a Pre Bariatric Surgical Intervention in High-Risk Patients: a Feasibility Study

**DOI:** 10.1007/s11695-018-3322-9

**Published:** 2018-06-09

**Authors:** Hafsa Younus, Saurav Chakravartty, Diwakar R. Sarma, Ameet G. Patel

**Affiliations:** 1 Department of Minimal Access Surgery, King’s College Hospital NHS Foundation Trust, Denmark Hill, London, SE5 9RS UK; 2 Ashford and St Peter’s Hospital, Kent, UK; 3 University Hospital North Midlands, Stoke-on-Trent, UK

**Keywords:** High risk, Super-obese, Endobarrier, OS-MRS, Roux-en-Y gastric bypass, Sleeve gastrectomy, ITU, Clavien-Dindo classification, Complications, Comorbidities

## Abstract

**Background:**

Obesity surgery mortality risk scoring system (OS-MRS) classifies patients into high, intermediate and low risk, based on age, body mass index, sex and other comorbidities such as hypertension and history of pulmonary embolism. High-risk patients not only have a higher mortality but are more likely to develop post-operative complications necessitating intervention or prolonged hospital stay following bariatric surgery. Endoscopically placed duodenal-jejunal bypass sleeve (Endobarrier) has been designed to achieve weight loss and improve glycaemic control in morbidly obese patients with clinically proven effectiveness.

The aim of this study was to assess if pre-operative insertion of endobarrier in high-risk patients can decrease morbidity and length of stay after bariatric surgery.

**Materials and Methods:**

Between 2012 and 2014, a cohort of 11 high-risk patients had an Endobarrier inserted (E&BS group) for 1 year prior to definitive bariatric surgery. These patients were compared against a similar group undergoing primary bariatric surgery (PBS group) during same duration. The two groups were matched for age, gender, body mass index, comorbidities, surgical procedure and OS-MRS using propensity score matching. Outcome measures included operative time, morbidity, length of stay, intensive therapy unit (ITU) stay, readmission rate, percentage excess weight loss (%EWL) and percentage total weight loss (%TWL).

**Results:**

Patient characteristics and OS-MRS were similar in both groups (match tolerance 0.1). There was no significant difference in total length of stay, readmission rate, %EWL and %TWL. Operative time, ITU stay, post-operative complications and severity of complications were significantly less in the E&BS group (*p* < 0.05) with significant likelihood of planned ITU admissions in the PBS group (*p* < 0.05).

**Conclusion:**

Endobarrier could be considered as a pre bariatric surgical intervention in high-risk patients. It may result in improved post-operative outcomes in high-risk bariatric patients.

## Introduction

The prevalence of morbid obesity has increased worldwide. Bariatric surgery has proven to be the most efficient method of maintained weight loss; however, it is not without risks [1–3]. Super-obese patients (BMI > 50 mg/kg^2^) are at higher risk of post-operative complications; this may be related to the presence of associated comorbidities such as type 2 diabetes, hypertension, ischaemic heart disease and obstructive sleep apnoea [4]. A validated scale to predict post-operative risk is obesity surgery mortality risk score (OS-MRS) which classifies patients into low-, intermediate- and high-risk groups based on the presence of risk factors [5, 6] [Table 1]. High-risk patients not only have a higher mortality but are more likely to develop post-operative complications necessitating intervention or prolonged hospital stay following bariatric surgery [4]. Pre-operative weight reduction may reduce morbidity and mortality in these patients undergoing bariatric surgery [7, 8].

**Table 1:** Obesity surgery mortality risk scoring system (OS-MRS)

Risk factor	Points	Score interpretation
1. Body mass index >50 kg /m^2^	1	0 to 1: class A—lowest risk (Mortality 0.31%)
2. Male sex	1	
3. Hypertension	1	2 to 3: class B—intermediate risk (Mortality 1.90%)
4. Risk for pulmonary embolism	1	
5. Age > 45 years	1	4 to 5: class C—high risk (Mortality 7.56%)
Total score	0–5	

Different strategies including diet, exercise programmes, low and very low calorie diets and pharmacotherapy have been devised to aid weight loss with minimal or no effect [8, 9]. Endobarrier is a non-surgical duodenal-jejunal bypass sleeve that has been developed to emulate the Roux-en-Y gastric bypass. It allows the transit of gastric secretions through to the jejunum without contact with duodenal wall and pancreatic enzymes resulting in change of metabolic parameters and glycaemic control by influencing key gut hormones including PYY and GLP-1 [10, 11]. These hormones are considered to play a key role in the pathogenesis of obesity; however, these changes are transient and are not sustained after explantation of the Endobarrier [12].

The aim of this feasibility study was to assess if the insertion of the Endobarrier in super-obese high-risk patients prior to definitive bariatric surgery could decrease morbidity and length of stay in this cohort of patients.

## Materials and Methods

### Study Design

This was a single-centre, single-surgeon, prospective case-control observational study of high-risk bariatric patients who underwent Endobarrier insertion during 2012–2014. EndoBarrier® (GI Dynamics, Boston, MA) use was approved by the Local New Clinical Procedure Committee (Institutional Review Board).

### Participants

Indication for bariatric surgery included compliance with established IFSO guidelines [13]. A cohort of 11 high-risk patients with BMI > 50 kg/m^2^ were enrolled in the study and had the Endobarrier inserted, followed by removal of the device, and definitive bariatric surgery (E&BS group). Patients had their obesity surgery mortality risk scoring system (OS-MRS) calculated in addition to other parameters outlined below.

The E&BS group were matched 1:1 with another group of high-risk bariatric patients (a cohort of 550 patients) who underwent primary bariatric surgery only (PBS group) during the same time interval. The case-control matching was performed using propensity score calculation with tolerance of 0.1, according to the type of surgery, comorbidities and OS-MRS. Gender, age and BMI were matched as part of OS-MRS to avoid duplication.

The presence of a comorbidity was established from the past medical history and pre-operative investigations. Improvement in comorbidity was considered if there was reduction or resolution in the pre intervention medication or therapy.

### Procedure

A 60-cm-long impermeable retrievable Endobarrier was delivered via endoscopy. A guide wire was placed into the duodenum over which the Endobarrier sleeve was deployed. Endobarrier sleeve was anchored in duodenum and advanced to terminate in the proximal jejunum.

The Endobarrier was retrieved 52 weeks later by a custom retrieval system and patients underwent definitive bariatric surgery as soon as possible.

Surgical procedures such as sleeve gastrectomy and gastric bypass are well described [14, 15]. Sleeve gastrectomy was performed by using a 38 Fr bougie and transaction at 2–4 cm from the pylorus. Gastric bypass was performed anticolic gastrojejunostomy with a 30-mL gastric pouch, 150-cm alimentary limb and 70-cm biliopancreatic limb.

### Post-operative Care

Post Endobarrier insertion, patients were started on fluid diet on the same day and followed up in dedicated research clinic at 6 weeks and 3, 6, 9 and 12 months. Post bariatric surgery patients were started on fluid diet on the first post-operative day and were discharged as soon as they were deemed suitable. They were seen in the dedicated research clinic at 6 weeks and 3, 6, 9 and 12 months.

### Parameters

Data collected for the E&BS group included combination of Endobarrier phase (from insertion to removal) and definitive bariatric surgery. This was then compared with the same data collected from the PBS group. Both groups were monitored for operative time, length of stay in intensive therapy unit (ITU), total length of stay, post-operative complications and severity of complications, readmission rate, percentage excess weight loss (%EWL), percentage total weight loss (%TWL) and improvements in comorbidities. ITU admission was decided by blinded anaesthetists based on institution protocol. Percentage excess weight loss was calculated as the weight of an individual in excess of their weight at a body mass index of 25 kg/m^2^. Post-operative complications were assessed according to Clavien-Dindo classification [16].

Cost-benefit analysis was carried out by evaluating total cost of all therapeutic interventions and treatments received by individual patients. Cost analysis included ward charges, cost for therapy, theatre and surgical equipment costs, anaesthesia costs, ITU/HTU cost, medication cost and medical staff costs for individual patient.

### Statistical Analysis

The results are expressed as the median ± range. Obesity-related comorbidities included diabetes, hypertension and obstructive sleep apnoea. Accuracy of matching was tested using Mann Whitney’s *U* test for non-normal distribution and chi-square test for categorical parameters. The two groups were compared for operative time, total length of stay, severity of complications, readmission rate and percentage excess weight loss using Wilcoxon rank test for matched pair analysis. All categorical data were compared using chi-square test and expressed in terms of odds ratio and confidence intervals for planned ITU stay and post-operative complications. The threshold for statistical significance was set at a *p* value of less than 0.05. All analysis was carried out by using SPSS statistical package version 23 (IBM Corp., Armonk, NY, USA).

## Results

Patient characteristics were similar in both the E&BS group and PBS group with regard to gender, age, BMI, comorbidities, OS-MRS and type of surgical intervention [Table 2].

**Table 2:** Patient characteristics

Parameters	E&BS group (*n* = 11)	PBS group (*n* = 11)	*p* value
Gender (M:F)	7:4	7:4	1
Age (years)	46 (34–68)	48 (36–66)	1
BMI (kg/m^2^)	61 (50–70)	58 (51–85)	0.8
Weight (kg)	172 (120–237)	172 (125–240)	1
Obesity-related comorbidities per patient	4 (1–5)	3 (2–5)	0.9
Diabetes	7/11	6/11	1
Hypertension	8/11	7/11	1
Obstructive sleep apnoea	8/11	8/11	1
Restricted mobility	8/11	6/11	1
Medication			
Anti-diabetic drugs*	2 (0–4)	1 (0–4)	0.6
Anti-hypertensive drugs*	2 (0–4)	2 (0–6)	0.9
CPAP use (number of patients)	6/11	5/11	1
OS-MRS	4 (2–5)	4 (2–5)	0.8
Type of surgery			1
Laparoscopic Roux-en-Y gastric bypass	1	1	
Laparoscopic sleeve gastrectomy	10	10	

*Number of drugs taken by each patient

*OS-MRS*, obesity surgery mortality risk scoring system; *LRYGB*, laparoscopic Roux-en-Y gastric bypass; *LSG*, laparoscopic sleeve gastrectomy

### Endobarrier Phase

Operative time for endobarrier insertion was a median of 30 min (range 11–45 min). Two patients developed post-operative nausea and vomiting, resulting in median total in hospital stay of 1 day (range 1–7). The Endobarrier remained in situ for a median of 52 weeks (range 43–59). There were no major adverse events, early explantations or readmissions during this period. Time from removal of Endobarrier to definitive bariatric surgery was 23 weeks (range 10–64).

**Fig. 1:**
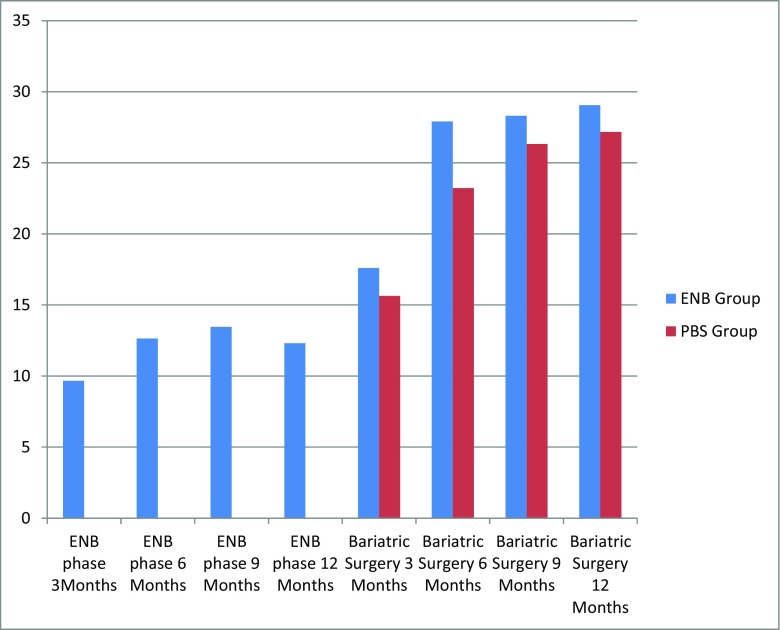
Median % total weight loss (%TWL) between the E&BS group and PBS group. %TWL is expressed on *y*-axis and duration of intervention is expressed on *x*-axis

**Fig. 2:**
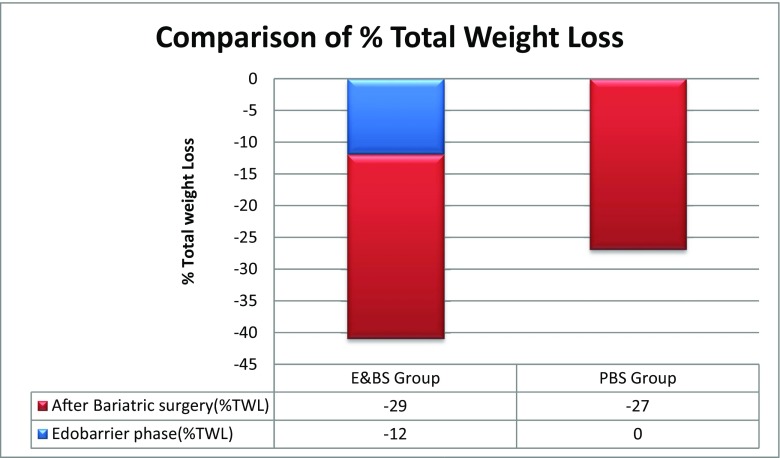
Comparison of %TWL between the E&BS group and the PBS group at 12 months following Endobarrier and bariatric surgery

Endobarrier insertion achieved a median %EWL of 16, 23, 24 and 19% at 3, 6, 9 and 12 months respectively. Similarly, %TWL was 10, 13, 13 and 12% at 3, 6, 9 and 12 months respectively [Figs. 1 and 2]. There was statistically significant reduction in HbA1c in diabetic patients from baseline 7.6 to 6.7 at 12 months post Endobarrier insertion (*p* < 0.05). Seven patients had type 2 diabetes and four patients had improvement of diabetes with two remissions. There was improvement of hypertension, obstructive sleep apnoea and mobility as a result of weight loss and two patients had reduction in OS-MRS [Table 3].

**Table 3:** Effect of the Endobarrier

	At baseline	At 12 months	*p* value
BMI (kg/m^2^)	61 (50–70)	58 (43–64)	*< 0.05*
Weight (kg)	172 (120–237)	158 (109–183)	*< 0.05*
HbA1C	7.6 (6.4–10.6)	6.7 (5.7–8.2)	*< 0.05*
Patients with reduction/cessation of medication			
Anti-diabetic drugs		4/7	
Anti-hypertensive drugs		2/8	
CPAP use reduction		1/6	
Improved mobility		6/8	

Data is presented as median (range). Comorbidity improvement is presented as reduction or improvement/total number of patients with comorbidity, Significant difference is presented in the form of italicised *p* value < 0.05.

### Endobarrier and Surgery vs. Primary Bariatric Surgery

LSG and LRNYGB were performed in equal proportion in both the E&BS and PBS groups (10:1) and several differences were seen in the peri-operative measures [Table 4]. Total operative time for the E&BS group was significantly higher compared to that for the PBS group (median 149 vs. 132 min, *p* < 0.05). Total operative time for the E&BS group included Endobarrier insertion time, removal time and time taken for definitive bariatric surgery. On the contrary, operative time for bariatric surgery alone was significantly lower in the E&BS group as compared to the PBS group (100 vs. 132 min, *p* < 0.05).

**Table 4:** Comparison of Post-operative outcomes between matched E&BS group and PBS group

Parameter	E&BS group*	PBS group	*p* value	OR (95% CI)
Total operating time (min)	149 (118–197)	132 (81–170)	*< 0.05*	
ITU stay	0/11 (0%)	5/11 (45%)	*< 0.05*	*1.83 (1.06–3.1)*
Length of ITU stay (days)	0	1 (0–3)	*< 0.05*	
Complications**	0 (0–1)	1 (0–4)	*< 0.05*	
Total length of stay (days)	5 (3–10)	5 (3–23)	0.5	
Total cost (£)	9766 (5670–16,391)	8057 (1857–27,477)	0.8	

*E&BS includes data from the endobarrier phase and definitive bariatric surgery

**Complications using Clavien-Dindo classification

*ITU*, intensive therapy unit; all continuous data is presented as median and range. Significant difference is presented in the form of italicised *p* value < 0.05

The median intensive therapy unit (ITU) stay was significantly less in the E&BS group [0 days (0–0)] compared to the PBS group [1 day (0–3), *p* < 0.05], with significantly more likelihood of planned ITU admission in the PBS group (0/11 vs. 5/11, odds ratio 1.83, CI 1.06–3.1, *p* = 0.03). The post-operative complications were similar in both groups (2/11 vs. 5/11, E&BS and PBS groups respectively). However, the E&BS group had significantly less severe complications (Clavien-Dindo classification 0 (0–1) vs. 1 (0–4), *p* < 0.05). Overall, there was no significant difference in total length of stay or readmission rate. There were two readmissions in the PBS group. There was no significant difference between the two groups in terms of total cost (£9766 vs. £8058, E&BS and PBS groups, respectively).

Both the E&BS group and the PBS group had similar %TWL after bariatric surgery. Median %TWL in the E&BS group was 12, 17, 28, 28 and 29% compared to 9, 16, 23, 26 and 27% in the PBS group at 3, 6, 9 and 12 months respectively [Figs. 1 and 2]. The E&BS group showed overall significant improvement in HbA1c from 6.8 (5.6–10.8) at baseline to 6 (5–9) at 12 months (*p* < 0.05) and fasting blood glucose (mmol/L) from 5.9 (4–12.4) at baseline to 5.05 (4–10) at 12 months (*p* < 0.05). Similarly, the PBS group showed significant improvement in % HbA1c and fasting blood glucose (mmol/L), from 6.9 (5–11) at baseline to 5.75 (5–7) at 12 months and 5.6 (4–12) at baseline to 5.2 (4–6) at 12 months, respectively (*p* < 0.05).

## Discussion

The incidence of complications in super-obese patients has been reported to be fourfold higher compared to morbidly obese group (BMI < 50 kg/m^2^) and 80% of the post bariatric surgery deaths occur in super-obese bariatric patients [17]. A study evaluating 185,315 patients based on the Bariatric Outcomes Longitudinal Database identified BMI greater than 50 as the strongest predictor of mortality [18].

Pre-operative weight loss is considered essential in optimising the super-obese patients. A study suggested that high-risk bariatric patients (BMI > 50 kg/m^2^) who are able to achieve weight loss of 5–10% excess body weight prior to surgery have a higher probability of shorter length of stay at hospital with more rapid post-operative weight loss [19].

Commonly used strategies for pre-operative weight loss include low-calorie diets with lifestyle modification and gastric balloon insertion. Liver-shrinking diets or very-low-calorie diets can shrink the liver substantially [20, 21], reduce intra-abdominal fat and thereby improve peri-operative outcomes. However, a multicentre randomised trial of 298 patients reported that a 14-day very-low-calorie diet had almost no impact on surgery apart from decrease in the number of minor post-operative complications [22].

Air and fluid filled intra-gastric balloons (IGB) have also been tried to achieve weight loss in the super-obese patients. In a randomised multicentre trial of 115 patients, 55 patients with a BMI of 54 kg/m^2^ had IGB inserted for 6 months which led to a BMI loss of 2.8 kg/m^2^ but this did not improve the peri-operative outcomes after laparoscopic gastric bypass [23]. This magnitude of weight loss could be achieved with diet, behaviour and exercise regimes [24]. On the contrary, in this study, Endobarrier appears to favourably impact peri-operative outcomes and may be considered as a more suitable pre-operative optimisation tool in high-risk bariatric patients than diets or IGB.

Previous studies have shown that Endobarrier can result in %EWL of 11–22% at 3 months [10, 25–27], which can reach up to 47% at 1 year [28]. Tarnoff et al. highlighted the efficacy of the EndoBarrier as significantly better weight loss than diet alone at 12 weeks (22.1 and 5.3% respectively) [29]. Our study showed maximum benefit at 9 months with median %EWL of 24.2% (%TWL 13.46%). In addition, our study as well as other studies showed that more than 90% of the endobarrier patients achieved at least a 10% EWL, compared to only 21–48% with diets and intensive weight loss counselling [30]. It is known that 5–10% weight loss is highly beneficial for cardiovascular risk reduction [7].

Weight loss improves insulin resistance by increasing hepatic insulin sensitivity and decreasing endogenous glucose production [31, 32]. However, Endobarrier has an advantage over diet as it can improve diabetic control even before significant weight loss. Our study showed that HbA1c significantly improved after endobarrier insertion. Previous studies showed that increased GLP-1 seen after bypass can also be seen as early as 24 h after endobarrier insertion and this effect is sustained till 1 week after explantation [33]. This may explain why there has been reduction and cessation of anti-diabetic medication in our studied group. Other randomised control trials have demonstrated a reduction or cessation of anti-diabetic medication at 3 and 12 months [27, 34]. One of the largest series aimed at diabetic patients (*n* = 198) concluded that at 12 months after Endobarrier insertion, patient’s weight, HbA1c levels and anti-diabetic medication use are reduced significantly [33]. Similarly, a significant improvement of blood pressure control with reduction in anti-hypertensive medication was seen in our studied group. Impaired functional status has also been known as a factor for post-operative morbidity or mortality [35]. Our study demonstrated a significant improvement in mobility of six out of eight patients.

Median operative time was significantly different between both groups. Total median operative time was significantly higher in the E&BS group as it included time taken for endobarrier insertion and removal (149 vs. 132 min, *p* < 0.05), but when compared time to perform bariatric surgery alone, median operative time was significantly lower in the E&BS group compared to the PBS group (100 vs. 132 min, *p* < 0.05). This might be due to reduced liver size, visceral fat and improved visibility as a result of pre-operative weight loss in the E&BS group [20, 21]. However, the median OS-MRS score did not change significantly after 12 months of Endobarrier insertion; two patients had reduction in OS-MRS (from 3 to 2 and from 5 to 3, respectively).

Stay in intensive therapy unit (ITU) has been used in the past as an endpoint to mark the severity of patient condition [24]. Our study demonstrated that ITU stay was significantly less in the E&BS group with significantly more likelihood of planned ITU admissions in the PBS group. A study involving 884 subjects suggested that high-risk bariatric patients (BMI > 50 kg/m^2^) who are able to achieve weight loss of 5–10% excess body weight prior to surgery have a higher probability of shorter length of stay at hospital with more rapid post-operative weight loss [19] but in our studied cohort, there was no significant difference in overall length of stay; however, our length of stay for the E&BS group included both endobarrier phase and bariatric surgery phase, thus prolonging total length of stay for ENG group. There was no significant difference between the two groups in terms of cost-benefit analysis; the E&BS group required more hospital visits, general anaesthesia, device and additional procedure-related costs, while the PBS group needed additional treatments for severe complications, prolonged ITU stay and increased nursing care costs.

The Endobarrier appears to be safe to use in our cohort. The recent US multi-centre ENDO Trial randomising obese patients with type 2 diabetes to either DJBS or sham was prematurely terminated as the incidence of liver abscesses (3.5%) exceeded a pre-defined safety threshold of 2%. Reassuringly, there were no major adverse effects in our study group. All patients were compliant and there was no need for early explantation in this cohort. More importantly, severity and risk of complication after definitive bariatric surgery were significantly less in patients who had pre-operative Endobarrier insertion. BMI (> 55 kg/m^2^), diabetes, hypertension, poor functional status, obstructive sleep apnoea and liver disease all contribute to greater mortality after bariatric surgery [18, 36]. Endobarrier can lead to optimisation of the comorbidities of the patient preparing for bariatric surgery. This is as important as weight loss parse. Unfortunately, these beneficial effects are transient after removal of the endobarrier; within a year, weight and HbA1c return to near baseline [12], hence making it suitable for pre-operative risk reduction. Our study has few limitations. The sample size was small and we did not study a diet weight loss-matched control group; further studies with larger group of patients are required.

This feasibility study shows that pre-operative Endobarrier insertion effectively reduces weight and improves glycaemic control, resulting in improved post-operative outcomes in high-risk bariatric patients. These results show that the endobarrier is a safe option for transient optimisation prior to bariatric surgery in super-obese high-risk bariatric patients.
